# FCP-Former: Enhancing Long-Term Multivariate Time Series Forecasting with Frequency Compensation

**DOI:** 10.3390/s25185646

**Published:** 2025-09-10

**Authors:** Ming Li, Muyu Yang, Shaolong Chen, Huangyongxiang Li, Gaosong Xing, Shuting Li

**Affiliations:** School of Computer Science and Technology/School of Artificial Intelligence, China University of Mining and Technology, Xuzhou 221116, China; lmgyw@cumt.edu.cn (M.L.); ts24170047a31@cumt.edu.cn (S.C.); huang_march@163.com (H.L.); xgs@cumt.edu.cn (G.X.);

**Keywords:** multivariate time series forecasting, frequency compensation layer, FCP-Former, transformer, patch

## Abstract

Long-term multivariate time series forecasting is crucial for real-world applications, including energy consumption, traffic flow, healthcare, and finance. Usually, some statistical approaches are used for predicting future observations based on historical temporal data. Recently, transformer-based models with patch mechanisms have demonstrated significant potential in enhancing computational efficiency. However, their inability to fully capture intra-patch temporal dependencies often limits the accuracy of predictions. To address this issue, we propose the Frequency Compensation Patch-wise transFormer (FCP-Former), which integrates a frequency compensation layer into the patching mechanism. This layer extracts frequency-domain features via Fast Fourier Transform (FFT) and incorporates them into patched data, thereby enriching patch representations and mitigating intra-patch information loss. To verify the feasibility of this model, FCP-Former was conducted on eight benchmark datasets via PyTorch 2.4.0 and trained on an NVIDIA RTX 4090 GPU (Santa Clara, CA, USA). Results demonstrate that FCP-Former 48 optimal experiment results and 17 suboptimal experiment results across all datasets. Especially on the ETTh1 dataset, it achieves an average MSE of 0.437 and an average MAE of 0.430, while on the Electricity dataset, it achieves an average MSE of 0.186 and an average MAE of 0.277. This demonstrates that FCP-Former has better forecasting accuracy and a superior ability to capture periodic and trend patterns.

## 1. Introduction

Time series forecasting constitutes a statistical approach aimed at predicting future observations based on historical temporal data. This methodology has demonstrated extensive applicability across a broad spectrum of domains, including but not limited to meteorology [1,2,3], healthcare analytics [4,5,6,7], intelligent transportation systems [8,9,10,11], electrical load forecasting [12,13,14,15], financial risk assessment [16,17,18,19], and Earth sciences [20,21]. In recent years, recurrent neural network (RNN)-based architectures have been extensively employed for modeling time series data due to their capacity to learn temporal dependencies [22,23]. While these methods have yielded considerable empirical success [24,25], they are inherently constrained by several limitations, most notably the issues of vanishing and exploding gradients. These challenges significantly hinder the ability of RNNs to effectively model long-range dependencies within sequential data, thereby limiting their performance in scenarios requiring long-term forecasting accuracy.

After achieving great success in computer vision [26,27,28,29] and natural language processing [30,31,32,33], the Transformer [34] model was introduced to time series forecasting to directly model the relationships between any two time steps in a sequence. Due to its powerful attention mechanism, the transformer overcomes the gradient vanishing and gradient exploding problems that still trouble RNN and LSTM (Long Short-Term Memory)-type methods, making it a popular research topic in the field of time series forecasting [35,36].

Based on the token granularity fed into the attention mechanism in the time domain, existing Transformer-based research can be roughly divided into patch-wise models and point-wise models. Point-wise models treat each time step and its corresponding variates as a token, which gives them a stronger ability to capture internal temporal variations. Typical point-wise models include FEDformer [35], Informer [37] and Autoformer [38]. However, due to their high computational complexity, it is challenging for these models to capture long-term dependencies between time series data. In contrast, for patch-wise models, a patch is a basic module formed by concatenating multiple temporally contiguous time series data points. This enables the model to treat a patch as a token instead of treating each timestep as a token, significantly reducing the computational time. Based on different treatments of the variates, patch-wise models can be further divided into channel-independent strategy models and channel-dependent strategy models. Typical channel-independent strategy models include PatchTST [39], while channel-dependent strategy models include iTransformer [40], TimeXer [41], and Crossformer [42].

As summarized in Table 1, previous patch-wise time series forecasting models mainly focused on leveraging the patching mechanism to capture long-term dependencies. PatchTST [39] and iTransformer [40] embed each patch into a coarse token through a temporal linear projection, which leads to their inability to fully utilize the data within the patch, potentially compromising the accuracy of the final prediction [41]. Crossformer incorporates a cross-variable attention mechanism, assuming that features influence each other and leveraging historical dependencies across variables for forecasting. Similarly, TimeXer [41] introduces exogenous variables with a design concept comparable to the cross-attention mechanism in Crossformer. The proposed solutions in previous patch-wise methods were confined to the time domain, overlooking the frequency domain, where periodicity and trends of time series are often more effectively captured.

To address the information loss resulting from the model’s inability to fully utilize intra-patch data, this study proposes FCP-Former, an optimized version of PatchTST [39], which integrates corresponding frequency-domain information into the patched data to enhance forecasting accuracy. The main contributions of this paper are summarized as follows:

- This study introduces a frequency compensation layer that integrates frequency domain features into the patching mechanism of Transformer-based models. This layer applies Fast Fourier Transform (FFT) to each patch to extract spectral components, performs representation learning in the frequency domain, and then reconstructs enriched patch representations via inverse FFT. This approach effectively mitigates intra-patch information loss by capturing periodic and trend features that are often overlooked in purely time-domain patch embeddings.
- A cross-patch frequency fusion mechanism via overlapping patches is proposed. By using overlapping patch segmentation with reduced stride, the model effectively integrates spectral information across adjacent patches. This enhances long-term periodicity and trend modeling. The fusion occurs within the frequency compensation layer, enriching patch tokens with broader contextual awareness without modifying the core attention structure.
- This study conducts extensive experiments on eight widely used benchmark datasets, demonstrating the superior performance of FCP-Former compared with state-of-the-art methods, and provides ablation studies and visual analyses to validate the effectiveness of the frequency compensation mechanism.

The remainder of this paper is organized as follows. Section 2 reviews the related work; Section 3 details the proposed FCP-Former; Section 4 validates experiments; Section 5 concludes.

## 2. Related Work

### 2.1. Problem Definition

Time series data is a set of data arranged in chronological order. This type of data is typically collected at specific time points, and there is a temporal dependence between the data points. In time series forecasting, future events are predicted by utilizing these time-ordered data. The historical data can be defined as $X_{t}=\{x_{1},x_{2},\ldots ,x_{L-1},x_{L}\}\in R^{L\times D}$ and the predicted data can be defined as ${\hat{X}}_{t}=\{{\hat{x}}_{L+1},{\hat{x}}_{L+2},\ldots ,{\hat{x}}_{L+T-1},{\hat{x}}_{L+T}\}\in R^{T\times D}$, where *D* is the number of variables, L is the length of historical data, and T is the length of predicted data. The concept of time series forecasting can be expressed as follows:

(1) $$\begin{array}{c} {\hat{X}}_{t}=f\left(X_{t}\right)+\epsilon , \end{array}$$

where ${\hat{X}}_{t}$ is the predicted value, f is the forecasting function, $X_{t}$ are the historical values, and ϵ is the forecasting error.

### 2.2. Transformer-Based Time Series Forecaster

With the great success made in the field of natural language processing and computer vision, Transformer has gained the attention of researchers in the field of time series forecasting due to its powerful ability to capture long-term temporal dependencies and complex multivariate correlations. This study briefly reviews several key variants below. Informer [37] addresses the high computational complexity of transformers in time series forecasting by proposing a sparse self-attention mechanism. FEDformer [35] enhances the transformer model’s ability to capture global features of time series data by combining the transformer model with seasonal trend decomposition while retaining key frequency information of the time series data through Fourier and wavelet transforms. PatchTST [39] improves the transformer’s ability to capture historical dependencies by using a channel-independent strategy to patch the time series data, reducing computational overhead while maintaining the ability to model long-range dependencies. Crossformer [42] enhances the transformer’s ability to handle multivariate time series forecasting tasks through dimension-segment-wise embedding and a two-stage attention mechanism. Npformer [43] introduces an innovative multi-scale segmented Fourier attention mechanism to more effectively capture dependencies. TimeXer [41] enhances the Transformer model’s prediction accuracy by incorporating exogenous variables. iTransformer [40] applies the Transformer’s attention mechanism along the variate dimension instead of the time dimension.

**Figure 1:**
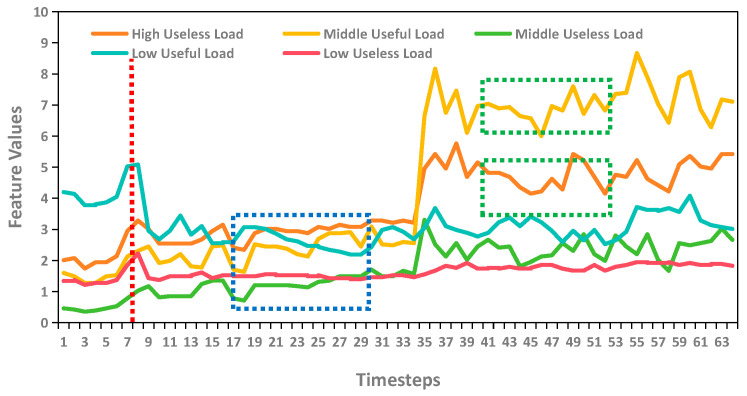
The difference in time steps between the point-wise model and the patch-wise model. The red dashed lines represent time steps from point-wise models, the blue rectangular areas represent time steps from patch-wise models using channel-dependent strategies, and the green rectangular areas represent time steps from patch-wise models using channel-independent strategies.

Most of these transformer-based models either focus on designing new attention mechanisms to reduce the complexity of the original attention mechanism or process the time series data itself to better leverage the transformer to achieve better performance on forecasting, especially when the prediction length is long. However, these patch-wise transformer methods face a common problem. As shown in Figure 1, compared to point-wise methods, the patch-wise approach, where the model treats a patch as a single token, cannot fully utilize each piece of data, which results in information loss within the patch. TimeXer introduces exogenous variables to address this issue. But the feature extracted from the time domain remains inherently limited. In contrast to TimeXer, this study utilizes the complementarity of frequency-domain information to time-domain data. The frequency compensation layer is proposed to extract features from the frequency domain, effectively overcoming the limitations of relying solely on time-domain feature extraction. This enables the model to better capture the periodic and trend characteristics of time series data.

### 2.3. Time Series Forecasting with Time–Frequency Analysis

The Fourier transform serves as a bridge for converting signals between the time and frequency domains, with the discrete Fourier transform (DFT) and discrete wavelet transform (DWT) commonly used tools for time–frequency analysis. Current mainstream time–frequency analysis methods can be categorized into two types. The first type involves transforming time-domain data into the corresponding Fourier spectrum, analyzing the Fourier spectrum to extract frequency-domain-based features, and then using inverse transformations to convert the data back to the time domain to obtain prediction results. Typical examples include FreTS [44], FITS [45], and SparseTSF [46]. In contrast, the second type simultaneously extracts features from both the time and frequency domains of time series data, with the extracted features then concatenated at the network output to produce the prediction result. A typical example is FEDformer [35]. The method proposed in this paper primarily addresses the issue of data loss within patches in patch-wise models. Since the first type of time–frequency analysis typically demands relatively low resource overhead, the proposed FCP-Former adopts this approach.

## 3. FCP-Former Principle

**Figure 2:**
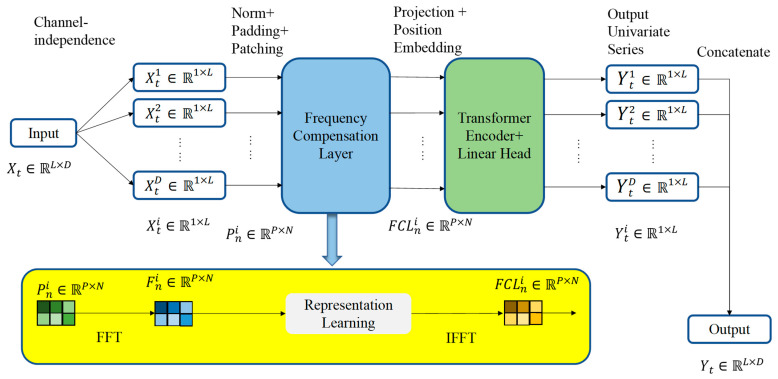
FCP-Former structure. The green rectangle in the yellow background represents time-domain data, the blue rectangle represents frequency-domain data, and the brown rectangle represents time-domain data with frequency-domain features. A channel-independent strategy and a frequency compensation layer are used to perform representation learning in the frequency domain for each patch. The ellipsis represents data from other independent feature channels. After representation learning is completed, the frequency compensation layer will fuse the frequency-domain features between patches, creating new patches with frequency-domain characteristics as learned data. The learned data is then converted back to the time domain via an inverse Fourier transform for embedding operations. The vanilla transformer encoder and linear layers are used to produce the prediction results.

As illustrated in Figure 2, FCP-Former includes three components: a patching module, a frequency compensation layer, and an encoder. Lu Han et al. [47] demonstrated through extensive experiments that the prediction method using channel-independent strategies typically achieves better prediction results than the method using channel-dependent strategies. Therefore, similar to PatchTST [39], FCP-Former adopts the channel-independent strategy. Furthermore, FCP-Former applies the frequency compensation layer to process the patched data before encoding, adding corresponding frequency features to compensate for intra-patch information loss. iTransformer [40] demonstrates that the standard attention mechanism can also yield excellent results. Therefore, instead of modifying the attention mechanism, FCP-Former focuses on enriching the information within each patch. Moreover, FCP-Former can adjust the patch length and the step size for dividing patches so that adjacent patches overlap. This overlapping portion serves as a bridge to fuse the frequency-domain features of different patches, further enhancing the model’s ability to capture the periodicity and trend of time series data.

### 3.1. Model Structure

FCP-Former consists of a patching module, a frequency compensation layer, and an encoder.

*Patching*: Following a channel-independent strategy, this study divides the original time series into D channels according to the data dimension and applies patching separately to each channel. The time series in the *i*-th channel is denoted as $X_{t}^{i}=\{x_{1}^{i},x_{2}^{i},\ldots ,x_{L-1}^{i},x_{L}^{i}\}\in R^{1\times L}$, where *L* is the sequence length. Let the patch length be *P*, the patch step size be *S*, and the number of patches be N, which is computed as follows:

(2) $$N=\left\lceil \frac{L-P}{S}\right\rceil +1,$$

Padding is applied at the end of the series. When the final patch extends beyond the sequence length, the remaining positions are filled with the last observed value $x_{L}^{i}$, ensuring that all patches have consistent size. After patching, the original time series in each channel is transformed into a sequence of patches $P_{n}^{i}=\{p_{1}^{i},p_{2}^{i},\ldots ,p_{N-1}^{i},p_{N}^{i}\}\in R^{P\times N}$.

**Figure 3:**
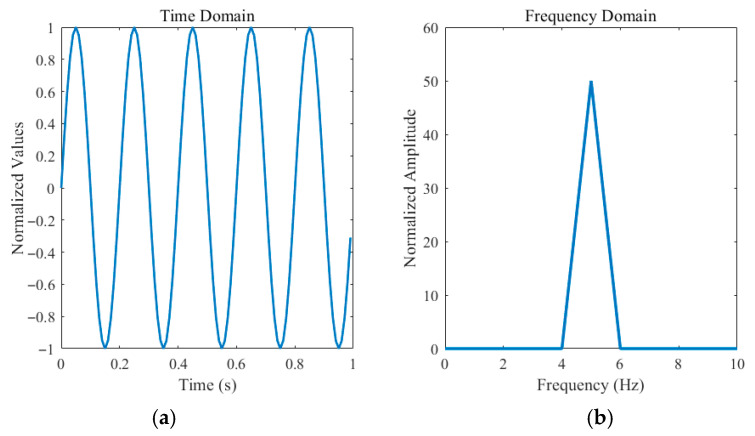
Data points in different domains. (**a**): Time-domain plots of a sine wave with a frequency of 5 Hz; (**b**) Frequency-domain plots of a sine wave with a frequency of 5 Hz.

*Frequency Compensation Layer*: As shown in Figure 3, in the time domain, data points are arranged in chronological order, with each data point representing the observation at a specific point in time. In the frequency domain, the data is decomposed into different frequency components, with each frequency component representing the extent of a particular periodicity in the signal. This representation facilitates the identification of periodicity and underlying trends in the data.

The core function of the frequency compensation layer is to perform representation learning on each patch in the frequency domain, thereby supplementing the temporal information that may be overlooked within individual time steps of the patch. Specifically, the frequency compensation layer first applies a Fast Fourier Transform (FFT) to the patch, converting the data into its frequency-domain representation. Representation learning is then conducted in the frequency domain, after which an inverse Fourier transform is applied to map the data back into the time domain. The processed patch thus represents an enriched version of the original data, combining both time-domain characteristics and frequency-domain features such as periodicity and trends. By incorporating spectral information, the frequency compensation layer effectively mitigates intra-patch information loss and enhances the model’s ability to capture fine-grained details within each patch, ultimately improving prediction accuracy. This study analyzes the frequency compensation layer in detail in Section 3.2.

*Encoder*: This study uses a vanilla Transformer encoder to map the patches processed by the frequency compensation layer into latent representations. Each patch is embedded into a latent space of dimension D by applying a learnable linear projection matrix $W_{p}\in R^{D\times P}$ and position encoding $W_{pos}\in R^{D\times N}$, which serves as the input to the encoder. The embedding process is formulated as follows:

(3) $$FCL_{n}^{i}=FCL\left(P_{n}^{i}\right),$$

(4) $${IN}_{d}^{i}=W_{p}{FCL}_{n}^{i}+W_{pos},$$

where FCL is the frequency compensation layer, $FCL_{n}^{i}$ is the result obtained after applying the frequency compensation layer to each patch, and ${IN}_{d}^{i}\in R^{D\times N}$ is the embedded result used as the input of the encoder. Then the multi-head attention will transform them into query matrices $Q_{h}^{i}$, key matrices $K_{h}^{i}$, and value matrices $V_{h}^{i}$. The attention output $OUT_{h}^{i}\in R^{D\times N}$ is ultimately obtained through scaled dot product. The attention process can be simply formulated as follows:

(5) $$Q_{h}^{i}={\left(IN_{d}^{i}\right)}^{T}W_{h}^{Q},$$

(6) $$K_{h}^{i}={\left(IN_{d}^{i}\right)}^{T}W_{h}^{K},$$

(7) $$V_{h}^{i}={\left(IN_{d}^{i}\right)}^{T}W_{h}^{V},$$

(8) $${\left(OUT_{h}^{i}\right)}^{T}=\mathrm{Attention}\left(Q_{h}^{i},K_{h}^{i},V_{h}^{i}\right)=\mathrm{Softmax}\left(\frac{Q_{h}^{i}K_{h}^{i^{T}}}{\sqrt{d_{k}}}\right)V_{h}^{i},$$

where $W_{h}^{Q},W_{h}^{K}\in R^{D\times d_{k}}$ and $W_{h}^{V}\in R^{D\times D}$. After passing through BatchNorm layers and a feed-forward network, the final predicted result can be obtained from a linear layer. The encoder and attention mechanism effectively capture the dependencies among patches processed by the frequency compensation layer, including both the temporal characteristics of the patches and the integrated frequency-domain features.

### 3.2. Analysis of Frequency Compensation Layer

As depicted in Figure 2, the frequency compensation layer is divided into the following steps to process each patch.

(1) *Fast Fourier Transform (FFT)*: The Fourier Transform can decompose a signal in the time domain into a linear combination of a series of sine and cosine functions. Each sine and cosine function represents a specific frequency component of the signal. Thus, the Fourier transform can extract the frequency characteristics from time series data. For discrete signals, the Discrete Fourier Transform is used:

(9) $$X_{k}=\sum_{n=0}^{N-1}\;x_{n}e^{-j2\pi \frac{k}{N}n},$$

where $X_{k}$ is the complex value of the *k*-th frequency in the frequency domain; $x_{n}$ is the *n*-th sampling point of the time-domain signal; and *N* is the length of the signal. Similarly, the IDFT is defined as follows:

(10) $$x_{n}=\sum_{n=0}^{N-1}\;X_{k}e^{j2\pi \frac{k}{N}n}.$$

Equation $\left(9\right)$ shows that for a signal of length *N*, the computational complexity of the DFT is $O(N^{2})$. However, the Fast Fourier Transform (FFT) reduces the computational load by utilizing the symmetry and periodicity of the signal, breaking the computation into smaller parts, thus reducing its complexity to $O(N{log}_{2}\;N)$ and significantly improving efficiency.

When performing Fast Fourier Transform on time series data, how to select the appropriate sampling rate is an issue that must be addressed. Retaining all frequency components may inevitably introduce noise interference, while preserving only a portion of the frequencies may have a risk of missing some underlying trends in the data. FEDformer [35] demonstrates that real-world multivariate time series typically yield low-rank matrices after Fourier transform. This low-rank property implies that representing the time series by randomly selecting a fixed number of Fourier components is reasonable. Consequently, this study adopts random sampling as our sampling method and sets the number of modes as *M*. The specific approach is as follows: First, the time series within the patch is transformed from the time domain to the frequency domain, resulting in a Fourier coefficient matrix $A\in R^{a\times b}$ (where a represents the number of time series and b represents the total number of Fourier components). Since a channel-independent strategy is employed to process patches, a is fixed at 1, and b corresponds to the length of the time series data within the patch. Next, M Fourier components are randomly selected from all b Fourier components to construct a selection matrix $S\in \{0,1{\}}^{M\times b}$, where $S_{i,k}=1$ indicates the selection of the k-th component, and $S_{i,k}=0$ represents the non-selection of that component. Finally, through matrix operations $A^{\prime }=A\cdot S^{⊤}$, a sparse matrix $A^{\prime }\in R^{1\times M}$ is obtained, retaining only the selected components, which serves as the sampling result.

(2) *Representation Learning In The Frequency Domain*: After random sampling, the selected set of frequency indices is defined as $I=\{i_{1},i_{2},\ldots ,i_{m}\}.$ Next, this study defines two weight tensors, ${𝒲}^{\left(1\right)}\in C^{F\times N\times N\times M}$ and ${𝒲}^{\left(2\right)}\in C^{F\times N\times N\times M}$, where F is the number of features, N is the number of patches, and M is the number of selected frequency components. These tensors are initialized with random values and serve as learnable weights for frequency-domain transformations. The input patch tensor is $X\in R^{B\times V\times PL\times N}$, where *B* is the batch size, *V* is the number of features, and *PL* is the patch length. This study applies a Fast Fourier Transform (FFT) to the input tensor *X* along the *PL* dimension. A tensor $Y_{ft}\in C^{B\times V\times PL\times N//2+1}$ is defined to store the frequency domain data after the Fourier transform. Through representation learning, the frequency-domain features such as periodicity and trends within the patches will be extracted and preserved.
(3) *Inverse Fast Fourier Transform*: The processed frequency-domain data is mapped back to the time domain using the Inverse Fast Fourier Transform (IFFT). This process can be simply formulated as follows:

(11) $$X_{ft}=FFT\left(X\right),$$

(12) $${𝒲}_{i}={𝒲}_{i}^{\left(1\right)}+j\cdot {𝒲}_{i}^{\left(2\right)},i\in I,$$

(13) $$Y_{ft}=\sum_{i}\;\left(X_{ft}\cdot {𝒲}_{i}\right),$$

(14) $$X_{out}=IFFT\left(Y_{ft}\right),$$

where X is the input tensor, $X_{ft}$ is the result obtained by applying the Fourier transform to X, ${𝒲}_{i}$ represents the learned complex weight for frequency *i*. $Y_{ft}$ is the frequency domain output, and the final reconstructed output is $X_{out}\in R^{B\times V\times PL\times N}$.

Generally, through the frequency compensation layer, the newly generated patches not only contain the time-domain features of the original patches but also include the frequency-domain features of the original patches and the fused frequency-domain features from adjacent patches. This enables the new patches to retain the original time-domain features while exhibiting more prominent periodicity and trend characteristics.

## 4. Experiments and Discussion

To verify the effectiveness and generality of FCP-Former, this study conducted several comprehensive experiments on eight real-world long-term time series forecasting datasets, which are widely used in practical applications. To ensure a fair comparison with baseline methods that typically use shorter look-back windows, this study set the similar input length of 96 in FCP-Former. This configuration abandons the potential advantage of longer look-back windows afforded by the patching mechanism and instead focuses on the inherent capabilities of the proposed frequency compensation layer. The results demonstrated that even under this constrained setting, FCP-Former obtained competitive performance in terms of MSE and MAE compared to existing state-of-the-art methods. Moreover, this study explored the performance of FCP-Former when utilizing longer look-back windows (336 and 512 input time steps), which demonstrated FCP-Former’s superior predictive capabilities.

### 4.1. Experimental Setup

#### 4.1.1. Datasets

The experiments utilized eight real-world datasets, which are widely applied in time series forecasting research. These datasets in detail are as follows:

ETT (Electricity Transformer Temperature): It contains two years of data from two different electricity transformers. ETTh1 and ETTh2 are recorded every hour, and ETTm1 and ETTm2 are recorded every 15 min.

Traffic: It contains data on hourly occupancy rates from 862 sensors on San Francisco Bay Area freeways from January 2015 to December 2016.

Weather: It includes 21 meteorological factors recorded every 10 min at the Weather Station of the Max Planck Biogeochemistry Institute in 2020.

Electricity: It records the hourly electricity consumption of 321 customers.

ILI: It describes the number of patients and influenza-like illness ratio at weekly intervals, obtained from the U.S. Centers for Disease Control and Prevention between 2002 and 2021.

The statistics of those datasets are summarized in Table 2.

#### 4.1.2. Baselines and Experimental Settings

This study chose the SOTA transformer-based model as the baseline, including PatchTST [39], iTransformer [40], TimeXer [41], FEDformer [35], Crossformer [42], and Autoformer [38]. All of the models follow the same experimental setup with prediction length T∈{24,36,48,60} for ILI dataset and T∈{96,192,336, 720} for other datasets. To validate the effectiveness of the model proposed in this study, this study adopted the multivariate time series forecasting setup from the TimeXer [41] study, setting the input length to 96. This setting is widely used in the literature to ensure a fair comparison across models while maintaining the generality of patch-based methods.

#### 4.1.3. Metrics

This study chose the mean square error (MSE) and mean absolute error (MAE) as evaluation metrics, which can be defined as follows:

(15) $$MSE=\frac{1}{N}\sum_{t=1}^{N}\;(Y_{t_{0}+t}-{\hat{Y}}_{t_{0}+t}{)}^{2},$$

(16) $$MAE=\frac{1}{N}\sum_{t=1}^{N}\;\left|Y_{t_{0}+t}-{\hat{Y}}_{t_{0}+t}\right|,$$

where *N* is the prediction length, $Y_{t}$ is the ground truth at timestamp t within the forecast horizon, and ${\hat{Y}}_{t}$ is the predicted value at timestamp t. A lower MSE or MAE indicates better forecasting performance.

#### 4.1.4. Implementation Details

FCP-Former was implemented with PyTorch 2.4.0 and trained on an NVIDIA GeForce RTX4090 GPU. This study used the Adam optimizer and set the learning rate to 1 × 10^−4^ to train the model. For small datasets, such as the ETT dataset, this study set the batch size to 128. For larger datasets like traffic, due to memory resource limitations, this study adjusted the batch size between 8 and 32. For all datasets, this study set the maximum number of training epochs to 50. To prevent overfitting and reduce training time, this study set the dropout rate to 0.05 and used an early stopping mechanism with a patience of 3 to halt training when the validation loss showed no significant decrease. The patch length, denoted as P, was set to 16. The hyperparameter frequency modes, denoted as M, were set to 16.

### 4.2. Experimental Results

As shown in Table 3, for multivariate forecasting, FCP-Former outperforms other methods on six datasets, excluding Traffic and Weather datasets. Specifically, on the ETTh1 dataset, FCP-Former achieves an average MSE of 0.437, outperforming the best baseline, PatchTST (0.460). For the ETTh2 dataset, FCP-Former records 0.365, which is lower than the second-best PatchTST (0.369) and significantly better than other baselines. For ETTm1 dataset, FCP-Former obtains 0.389, slightly ahead of PatchTST (0.390) and considerably lower than Crossformer (0.602). For the ETTm2 dataset, FCP-Former achieves 0.280, surpassing PatchTST (0.291) and other methods. For the large-scale Electricity dataset, FCP-Former reaches 0.186, the best result among all methods, outperforming the second-best PatchTST (0.198). Finally, on the ILI dataset, FCP-Former yields an average MSE of 1.734, which is lower than PatchTST (1.765) and substantially better than FEDformer (3.692) and Crossformer (2.740). On datasets with strong periodicity and trend components, such as ETT and Electricity, FCP-Former benefits from the frequency compensation layer, which makes it more sensitive to periodic and trend-related patterns, thereby enhancing forecasting accuracy. In contrast, baseline methods such as PatchTST and iTransformer suffer from limitations in fully exploiting intra-patch information due to their patching mechanisms, while TimeXer, Crossformer, and Autoformer remain confined to the time domain and thus fail to utilize the rich information available in the frequency domain. This limitation likely contributes to their comparatively lower forecasting accuracy. The experimental results indicate that FCP-Former significantly outperforms other baseline methods in prediction performance for multivariate long-term time series forecasting tasks. FCP-Former achieves a total of 48 optimal values and 17 suboptimal values, especially on the ETT and electricity datasets. Although FCP-Former does not achieve the best performance on all datasets, it consistently attains near-optimal results. This demonstrates that FCP-Former exhibits significant advantages in long-term forecasting. It is worth noting that, compared to baseline methods, FCP-Former often exhibits a smaller MAE when the MSE values are similar. For example, in ETTm2 dataset, the average MAE of FCP-Former is 0.323, while the suboptimal average MAE is TimeXer (0.326). This indicates smaller average deviations between predictions and ground truth, reflecting higher overall prediction accuracy. In practical application scenarios, such as stock price forecasting, supply chain management, and healthcare, where a lower MAE is more critical, FCP-Former demonstrates a distinct advantage. Additionally, this study observed that FCP-Former performs poorly on the Traffic and Weather datasets. This could be attributed to the noise in the data, where certain periodicities are less evident. For instance, the Traffic dataset contains not only locally periodic daily commuting data but also a substantial amount of vehicle pass data with no discernible periodicity. The design of the frequency compensation layer makes FCP-Former more sensitive to periodic and trend-based features. Therefore, the aforementioned noise limits its predictive performance in certain scenarios, preventing the model from achieving the desired forecasting accuracy. In contrast, on datasets with more pronounced periodicity and less noise, such as the ETT and Electricity datasets, the model demonstrates superior performance. This suggests that FCP-Former is better suited for applications with clear periodic patterns, particularly in industrial settings.

### 4.3. Model Analysis

This study analyzes FCP-Former performance ablation experiments, hyperparameter sensitivity experiments, different input lengths experiments, capture information ability experiments from each timestep, and robustness experiments.

#### 4.3.1. Ablation Experiments

In this section, this study conducted an ablation study on the model to demonstrate the effectiveness of the frequency compensation layer. The component w/o FCL is ablated, which means removing the frequency compensation layer before encoding.

This study compared the performance of the FCP-Former ablation version and the results of the full FCP-Former model in Table 4. On the ETTm2 dataset, FCP-Former achieves an average MSE of 0.280 and MAE of 0.323, representing 3.78% and 3.58% improvements compared with the model without FCL (0.291/0.335). On the Weather dataset, the average MSE and MAE decrease from 0.257/0.281 (w/o FCL) to 0.245/0.275, corresponding to 4.67% and 2.14% improvements, respectively. Similarly, on the Electricity dataset, FCP-Former achieves the lowest average MSE (0.186) and MAE (0.277), yielding 6.06% and 1.77% gains over the variant without FCL (0.198/0.282). From the results of the ablation experiment, it is evident that the application of the frequency compensation layer leads to improved prediction performance.

#### 4.3.2. Hyperparameter Sensitivity Experiments

**Figure 4:**
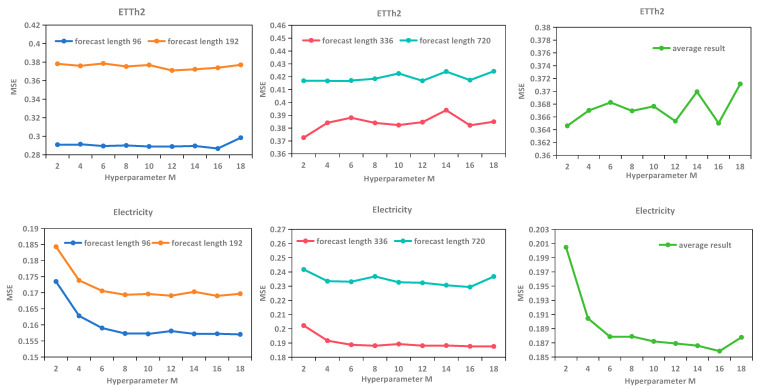
The impact of different hyperparameters M on MSE in the ETTh2 (**upper row**) and Electricity (**lower row**) datasets.

In the frequency compensation layer, this study employed a crucial hyperparameter: the number of modes in the frequency domain, M. This hyperparameter determines how many frequency components are selected from the frequency domain for the model to learn from. Its value directly impacts both the model’s frequency domain representation capability and computational complexity. Theoretically, a larger number of modes implies more frequency patterns are used, resulting in higher frequency domain resolution and finer data variations being captured, but at the cost of increased computational load and a higher risk of overfitting. On the other hand, a smaller number of modes compresses the frequency domain information, with the model focusing only on the main low-frequency components. This makes the model lighter and faster but may lead to the loss of high-frequency information, decreasing representational capacity while potentially improving generalization performance. In this experiment, this study set the patch length to 32 and evaluated the number of modes in the frequency domain M from the set {2, 4, 6, 8, 10, 12, 14, 16, 18}. The results are shown in Figure 4. This figure corroborates the aforementioned theoretical analysis. When the value of M is low, the model learns fewer frequency patterns, resulting in relatively lower prediction accuracy. As M increases, the MSE gradually decreases and plateaus. When M reaches 16, the model achieves its optimal performance for this hyperparameter on both the ETTh2 and Electricity datasets. However, as M continues to increase, the model’s prediction performance deteriorates due to overfitting, leading to a rise in MSE. This trend of performance deterioration due to overfitting is more pronounced on the Electricity dataset. Considering both computational costs and prediction performance, this study recommends setting the value of M to 16.

#### 4.3.3. Different Input Lengths Experiments

In time series forecasting tasks, the input length determines the amount of historical information available to the model. A longer look-back window allows the model to capture a broader range of past observations, thereby expanding its perceptual scope. Following the approach in the PatchTST [39] study regarding the selection of the look-back window size, this study designed two experimental models for this study: FCP-Former-336, with a look-back window length of 336, and FCP-Former-512, with a look-back window length of 512. Since a longer look-back window inevitably leads to increased memory overhead, this study dynamically adjusted the batch size to balance memory consumption. Due to the limited size of the ILI dataset, with only 966 data points, increasing the input length leads to a reduction in the training set size. For FCP-Former-512, using the dataset split as shown in Table 2, the training set consists of only eight data points, making training impossible. Similarly, for FCP-Former-336, the training set contains only 184 data points, which is insufficient for adequate model training. Therefore, this study did not conduct experiments on the ILI dataset. For the remaining datasets, the comparative results of FCP-Former (look-back window length of 96), FCP-Former-336, and FCP-Former-512 are presented in Table 5. For small-scale datasets, such as ETTh2, FCP-Former-512 achieves an average MSE of 0.342, compared with 0.365 for the vanilla model, while the MAE also improves from 0.395 to 0.392. For large-scale datasets, on the Weather dataset, FCP-Former-512 attains an average MSE of 0.226 and MAE of 0.270, both lower than those of the original model (0.245/0.275). Based on the work of Wang et al. [48], it is evident that due to the presence of repeated short-term patterns in the data and the difficulty of Transformer models in effectively capturing and modeling these short-term patterns, the performance of Transformer-based models often deteriorates as the input length increases. This phenomenon explains the performance of FCP-Former-336, which, in a few specific cases, marginally outperformed that of FCP-Former-512. Overall, the performance of FCP-Former-512 surpasses that of both FCP-Former-336 and FCP-Former, particularly when the prediction length is higher, where its advantages become even more pronounced. The overall superior performance of FCP-Former-512, particularly for longer prediction horizons, suggests that FCP-Former performs well in capturing long-term temporal dependencies and in deeply extracting meaningful information from historical data.

#### 4.3.4. Capture Information Ability Experiments from Each Timestep Within the Patches

Under a fixed-length look-back window, increasing the patch length in the patch-wise time series prediction model leads to a reduction in prediction accuracy. This is due to the model’s difficulty in capturing the information of each time step within the patch and its inability to extract more information from the look-back window. To investigate whether the method proposed in this study can effectively capture the information of each time step within the patch, this study conducted experiments by adjusting the patch length multiple times under a fixed look-back window. The dataset selected for the experiment is ETTh1, with PatchTST [39] baseline methods used for comparison. The look-back window is fixed at 96, and the patch lengths are set to 16, 24, and 32, respectively. In this experiment, the results with a patch length of 16 will be used as the baseline to investigate the impact of increasing the patch length on the MSE and MAE of the prediction results. The experimental results are shown in Table 6 and Table 7.

The results indicate that increasing the patch length from 16 to 24 has a negligible effect on FCP-Former’s prediction accuracy. For MSE, when the prediction lengths are 96 and 336, the MSE increases by only 0.26% and 2.12%, respectively, and there is almost no effect at 192 and 720. In contrast, PatchTST experiences a larger decline in prediction accuracy as the patch length increases. This decline becomes more evident at a patch length of 32, particularly for a prediction length of 720, where the MSE increase is only 1.06% for FCP-Former compared to 11.01% for PatchTST. A similar trend is observed for MAE. When the prediction lengths are 96 and 336, the MAE of FCP-Former increases by only 0.25% and 0.67%, respectively. By comparison, PatchTST shows a larger degradation, with the average MAE rising by 2.91%, whereas FCP-Former increases by only 0.46%. The difference becomes more pronounced at a patch length of 32, where for a prediction length of 720 the MAE increase is merely 0.43% for FCP-Former, in contrast to 5.21% for PatchTST. These findings demonstrate that patch-wise models generally lose accuracy due to their inability to capture each timestep, but FCP-Former effectively captures information from every time step within the patch.

#### 4.3.5. Robustness Experiment

Robustness is an essential metric for evaluating a model’s predictive stability. To assess the robustness of FCP-Former, this study conducted 10 independent predictions for forecasting horizons of 96, 192, 336, and 720 on the ETTh1 dataset. A 90% confidence interval was applied to determine whether FCP-Former’s predictions in Table 3 are reliable. The experimental results are shown in Table 8. From these results, it can be observed that the effect of training randomness on predictive performance is minimal, confirming that FCP-Former exhibits strong robustness.

### 4.4. Multivariate Showcases

**Figure 5:**
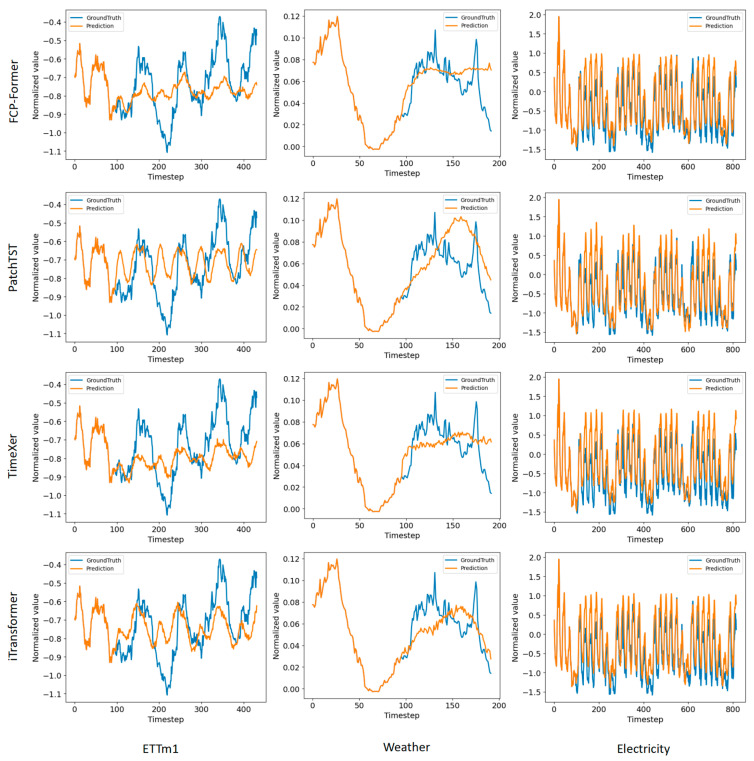
Visualization results of forecasting sequences randomly selected from ETTm1, Weather, and Electricity. The data alignment is based on the same time steps.

As illustrated in Figure 5, this study also compares the prediction results of FCP-Former with those of recently established state-of-the-art models (PatchTST, TimeXer, and iTransformer) on multiple datasets (ETTm1, Weather, and Electricity). The experimental setup in this study is consistent with the setup presented in Table 3, with an input length of 96. This implies that the first 96 points represent the input historical data, where predictions and ground truth are identical. The subsequent points represent forecasts, each aligned with a ground truth value for evaluation. FCP-Former demonstrated predictions that are closest to the ground truth values, providing a direct visual manifestation of its superior MAE performance reported in Table 3. Furthermore, due to its enhanced ability to capture the trend of time series data, FCP-Former exhibits a significant advantage in predicting the overall trend, as evidenced by the close alignment between its predicted trends and the actual trends. And in the later segments of the Weather dataset predictions, both FCP-Former and TimeXer produced relatively flat forecasts. This is likely due to the presence of noisy data within the retrospective window during the prediction of the latter part of the series. Both TimeXer and the proposed FCP-Former handle the ability to capture information within model patches, enhancing the capacity to capture patch-level information. The captured noise data somewhat interferes with the model’s ability to discern the trend of data variations, leading to flatter predictions. In contrast, PatchTST and iTransformer do not perform any special handling of patch-level data and are less influenced by noise. But PatchTST and iTransformer predict an upward trend at time step 150, while the true values exhibit a downward trend, resulting in slightly inferior overall performance compared to FCP-Former and TimeXer.

### 4.5. Training Costs Evaluation

The performance of current time series forecasting models is steadily improving. However, the excessive number of parameters and prolonged training times remain significant challenges. The proposed FCP-Former addresses patch data through the frequency compensation layer, adding only a minimal number of parameters to achieve an improvement in prediction performance. To specifically assess the impact of the frequency compensation layer on training overhead, this study analyzed the cost of FCP-Former, including runtime, GPU utilization, and performance in terms of MSE and MAE. Under the same experimental setup, this study compared the resource overhead of FCPformer with the baseline models mentioned in Table 3 on the ETTh1 dataset. In this experiment, this study set the prediction length to 96, the input length to 96, and the batch size to 32. “Iter” denotes the time required to train each iteration. To mitigate overfitting and reduce training time, this study implements an early stopping mechanism with a patience of 3, halting training when the validation loss exhibited no significant improvement. Consequently, different models have varying numbers of epochs, with a smaller number of epochs indicating a faster convergence rate of the model. The experimental results are presented in Table 9. For the ETTh1 dataset, the TSPE (Time Spent Per Epoch) and GPU usage of the patch-wise Transformer-based model are significantly smaller than those of the point-wise Transformer-based model.

**Figure 6:**
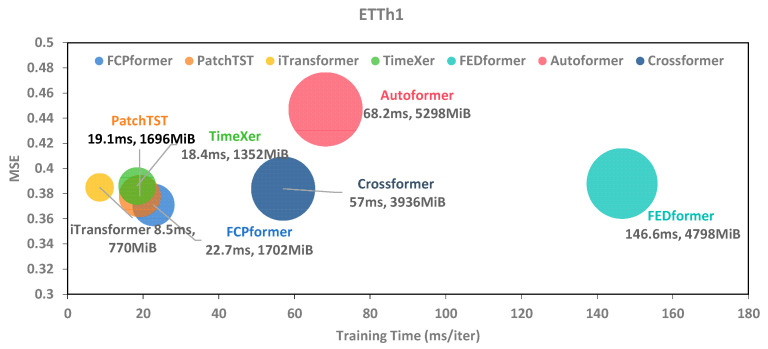
Comprehensive performance analysis with training time, metrics, and GPU occupancy in the ETTh1 datasets. A larger graph size indicates more GPU usage.

Compared to PatchTST, FCP-Former achieved better forecasting performance while incurring only minor resource overhead: an additional 6 MiB of GPU memory and 3.6 ms per iteration. This demonstrated that the improvement in model prediction performance due to the frequency compensation layer far outweighs the resource overhead it introduces. Notably, the training speed per epoch (TSPE) of the patch-wise Transformer model for time series forecasting is significantly smaller than that of the point-wise Transformer model for time series forecasting. In contrast, iTransformer employs a different approach, treating the entire time series of each variate as a single token and applying attention along the variate dimension. This architectural choice inherently minimizes the computational overhead associated with temporal patching. In addition, as shown in Figure 6, this study provided a more intuitive comparison of the training speed, GPU utilization, and performance across multiple models.

## 5. Conclusions

This study introduces FCP-Former, a time series forecasting method that enhances patch-wise Transformer models through a novel frequency compensation layer. This layer enables representation learning in the frequency domain, enriching the information within each patch and fusing frequency features across patches to improve the capture of periodic and trend components in long-term multivariate time series. Experiments on eight real-world datasets (ETT, Weather, Traffic, Electricity, ILI, etc.) show that FCP-Former achieves state-of-the-art performance, obtaining 48 optimal experiment results and 17 suboptimal experiment results in MSE and MAE. For instance, for ETTm1, it attains an average MSE of 0.389 and an MAE of 0.401, outperforming PatchTST and iTransformer. On Electricity, it scores an MSE of 0.186 and an MAE of 0.277, significantly improving upon FEDformer and Autoformer. However, performance is tied to data periodicity: FCP-Former excels on highly periodic, low-noise data like ETT and Electricity but lags on aperiodic or noisy datasets such as Traffic and Weather. For example, on Traffic, it is slightly surpassed by iTransformer. Limitations include reduced effectiveness on non-periodic data and sensitivity to noise. Future work will focus on improving adaptability to aperiodic signals via noise suppression mechanisms and enhanced non-periodic feature extraction, as well as optimizing computational efficiency for deployment in resource-constrained environments.

## Figures and Tables

**Table 1:** Summary of time series forecasting methods.

Type	Method	Approach	Data Domain	Train Speed	Gap
Patch-wise	PatchTST [39]	Patch mechanism	Time domain	Fast	Poor ability to capture internal information within the patch
	iTransformer [40]	Reverse dimension Patch mechanism	Time domain	Very fast	Poor ability to capture internal information within the patch
	TimeXer [41]	Exogenous variables	Time domain	Fast	Only captures internal information within the patch in the time domain
	Crossformer [42]	Cross-dimension attention	Time domain	Slow	Only captures internal information within the patch in the time domain
Point-wise	FEDformer [35]	Frequency-enhanced attention	Time–frequency domain	Very slow	High training overhead
	Informer [37]	Sparse self-attention	Time domain	Medium	High training overhead
	Autoformer [38]	Seasonal self-attention mechanism	Time domain	Slow	High training overhead

**Table 2:** Details of datasets.

Datasets	ETTh	ETTm	Traffic	Weather	Electricity	ILI
Timesteps	17,420	69,680	17,544	52,696	26,304	966
Features	7	7	862	21	321	7
Partitions (train/val/test)	12/4/4	12/4/4	7/1/2	7/1/2	7/1/2	6/2/2

**Table 3:** Multivariate long-term forecasting results. “96, 192, 336, and 720” respectively represent prediction lengths of 96, 192, 336, and 720. “avg” denotes the average of the results across these four prediction lengths.

Methods		FCP-Former		PatchTST		iTransformer		TimeXer		FEDformer		Crossformer		Autoformer	
Metric		MSE	MAE	MSE	MAE	MSE	MAE	MSE	MAE	MSE	MAE	MSE	MAE	MSE	MAE
ETTh1	96	**0.378**	**0.395**	**0.378**	**0.395**	0.385	0.404	0.386	0.399	0.388	0.425	0.384	0.408	0.447	0.451
	192	**0.426**	**0.421**	0.443	0.435	0.441	0.438	0.438	0.432	0.437	0.450	0.433	0.435	0.486	0.475
	336	**0.472**	**0.445**	0.493	0.461	0.479	0.456	0.483	0.455	0.482	0.476	0.677	0.628	0.505	0.490
	720	**0.471**	**0.460**	0.527	0.499	0.489	0.482	0.491	0.476	0.502	0.498	0.670	0.616	0.517	0.519
	avg	**0.437**	**0.430**	0.460	0.447	0.449	0.445	0.449	0.440	0.452	0.462	0.541	0.522	0.489	0.484
ETTh2	96	**0.287**	**0.339**	0.292	0.343	0.297	0.347	0.289	0.342	0.339	0.383	0.678	0.634	0.344	0.385
	192	0.374	**0.394**	0.373	0.399	0.378	0.398	**0.371**	**0.394**	0.414	0.427	1.141	0.745	0.422	0.433
	336	**0.382**	**0.412**	0.390	0.416	0.426	0.433	0.419	0.430	0.453	0.464	1.200	0.764	0.455	0.464
	720	0.417	**0.437**	0.422	0.443	0.430	0.448	0.416	0.438	0.480	0.487	1.384	0.836	0.465	0.477
	avg	**0.365**	**0.395**	0.369	0.400	0.383	0.407	0.374	0.401	0.422	0.441	1.101	0.745	0.421	0.440
ETTm1	96	**0.322**	**0.360**	0.330	0.367	0.360	0.387	0.330	0.367	0.373	0.419	0.343	0.381	0.620	0.528
	192	0.368	**0.386**	0.370	0.387	0.389	0.405	**0.367**	0.387	0.415	0.440	0.375	0.403	0.603	0.519
	336	0.399	**0.407**	**0.398**	0.411	0.419	0.416	0.401	0.411	0.450	0.460	0.413	0.424	0.622	0.526
	720	0.467	0.452	0.461	**0.444**	0.493	0.458	0.467	0.450	0.509	0.487	0.530	0.508	0.565	0.515
	avg	**0.389**	**0.401**	0.390	0.403	0.415	0.417	0.391	0.403	0.437	0.452	0.415	0.429	0.602	0.522
ETTm2	96	0.177	**0.257**	0.185	0.264	0.181	0.265	**0.175**	0.258	0.192	0.282	0.269	0.351	0.220	0.303
	192	0.240	**0.298**	0.247	0.307	0.250	0.310	**0.238**	0.300	0.264	0.324	0.363	0.419	0.272	0.330
	336	0.301	0.340	0.309	0.346	0.315	0.352	**0.296**	**0.339**	0.325	0.362	0.673	0.596	0.327	0.365
	720	**0.401**	**0.398**	0.422	0.422	0.411	0.406	0.405	0.406	0.421	0.416	2.652	1.111	0.421	0.418
	avg	0.280	**0.323**	0.291	0.335	0.289	0.333	**0.279**	0.326	0.301	0.346	0.989	0.619	0.310	0.354
Traffic	96	0.490	0.311	0.492	0.314	**0.427**	**0.289**	0.466	0.302	0.575	0.354	0.528	0.293	0.647	0.396
	192	0.486	0.307	0.482	0.305	**0.456**	0.305	0.485	0.317	0.647	0.406	0.544	**0.295**	0.666	0.418
	336	0.502	0.318	0.495	0.311	**0.476**	0.316	0.502	0.322	0.669	0.419	0.572	**0.298**	0.699	0.434
	720	0.537	0.335	0.528	0.330	**0.514**	0.341	0.538	0.340	0.721	0.444	0.596	**0.311**	0.710	0.440
	avg	0.504	0.318	0.499	0.315	**0.468**	0.313	0.498	0.320	0.652	0.420	0.560	**0.299**	0.680	0.422
Weather	96	0.162	0.209	0.175	0.217	0.173	0.211	**0.158**	**0.204**	0.220	0.299	**0.158**	0.235	0.253	0.323
	192	0.210	0.253	0.222	0.259	0.222	0.254	0.206	**0.250**	0.283	0.350	**0.203**	0.267	0.298	0.353
	336	0.265	0.293	0.276	0.298	0.281	0.298	0.263	**0.292**	0.347	0.399	**0.254**	0.309	0.357	0.394
	720	**0.343**	0.344	0.354	0.351	0.356	0.349	**0.343**	**0.343**	0.402	0.413	0.367	0.391	0.419	0.427
	avg	0.245	0.275	0.257	0.281	0.258	0.278	**0.242**	**0.272**	0.313	0.365	0.246	0.301	0.332	0.374
Electricity	96	**0.156**	**0.250**	0.167	0.254	0.158	0.252	0.162	0.252	0.215	0.327	0.219	0.314	0.207	0.321
	192	**0.169**	**0.262**	0.180	0.267	0.189	0.274	0.192	0.279	0.232	0.341	0.231	0.322	0.216	0.327
	336	**0.188**	**0.280**	0.198	0.284	0.208	0.294	0.208	0.295	0.254	0.359	0.246	0.337	0.271	0.368
	720	**0.229**	**0.317**	0.238	**0.317**	0.254	0.331	0.249	0.329	0.305	0.394	0.280	0.363	0.282	0.377
	avg	**0.186**	**0.277**	0.198	0.282	0.207	0.291	0.206	0.293	0.252	0.356	0.244	0.334	0.244	0.348
ILI	24	1.689	**0.803**	**1.650**	0.804	2.357	1.058	2.333	1.042	4.077	1.424	3.370	1.193	2.802	1.153
	36	**1.573**	**0.777**	1.714	0.853	2.236	1.027	2.192	0.976	3.865	1.414	3.533	1.219	2.734	1.085
	48	**1.684**	**0.815**	1.718	0.863	2.207	1.020	2.173	0.969	3.881	1.404	3.790	1.263	2.592	1.045
	60	1.992	**0.905**	**1.977**	0.934	2.212	1.036	2.111	0.961	3.947	1.409	4.076	1.327	2.833	1.127
	avg	**1.734**	**0.825**	1.765	0.863	2.253	1.035	2.203	0.987	3.943	1.413	3.692	1.250	2.740	1.102
SOTA counts		48		7		6		16		0		7		0	

The best results are in bold and the second best are underlined.

**Table 4:** Results of the ablation study of FCP-Former.

Methods			FCP-Former			w/o FCL	
Metric		MSE	|ΔMSE%|	MAE	|ΔMAE%|	MSE	MAE
ETTm2	96	**0.177**	4.32%	**0.257**	2.65%	0.185	0.264
	192	**0.240**	2.83%	**0.298**	2.93%	0.247	0.307
	336	**0.301**	2.58%	**0.340**	1.73%	0.309	0.346
	720	**0.401**	4.98%	**0.398**	5.69%	0.422	0.422
	avg	**0.280**	3.78%	**0.323**	3.58%	0.291	0.335
Weather	96	**0.162**	7.43%	**0.209**	3.69%	0.175	0.217
	192	**0.210**	5.71%	**0.253**	2.32%	0.222	0.259
	336	**0.265**	3.99%	**0.293**	1.68%	0.276	0.298
	720	**0.343**	3.11%	**0.344**	1.99%	0.354	0.351
	avg	**0.245**	4.67%	**0.275**	2.14%	0.257	0.281
Electricity	96	**0.157**	5.99%	**0.251**	1.18%	0.167	0.254
	192	**0.169**	6.11%	**0.262**	1.87%	0.180	0.267
	336	**0.188**	5.05%	**0.280**	1.41%	0.198	0.284
	720	**0.229**	3.78%	**0.317**	0%	0.238	**0.317**
	avg	**0.186**	6.06%	**0.277**	1.77%	0.198	0.282

The best results are in bold. |ΔMSE%| and |ΔMAE%| represent the percentage improvements in MSE and MAE performance, respectively, compared to the case without FCL.

**Table 5:** Multivariate long-term forecasting results with FCP-Former-336 and FCP-Former-512.

Methods		FCP-Former		FCP-Former-336		FCP-Former-512	
Metric		MSE	MAE	MSE	MSE	MSE	MAE
ETTh1	96	0.378	0.395	0.379	**0.400**	**0.376**	0.403
	192	0.426	**0.421**	**0.411**	0.422	0.421	0.439
	336	0.472	**0.445**	0.482	0.472	**0.438**	0.453
	720	**0.471**	**0.460**	0.505	0.500	0.475	0.484
	avg	0.437	**0.430**	0.444	0.448	**0.427**	0.445
ETTh2	96	0.287	**0.339**	0.290	0.349	**0.280**	0.343
	192	0.374	0.394	0.340	0.385	**0.331**	**0.383**
	336	0.382	0.412	**0.353**	**0.402**	0.361	0.407
	720	0.417	0.437	0.408	0.440	**0.395**	**0.434**
	avg	0.365	0.395	0.348	0.394	**0.342**	**0.392**
ETTm1	96	0.322	0.360	**0.296**	**0.350**	0.304	**0.350**
	192	0.368	0.386	**0.343**	**0.375**	0.345	**0.375**
	336	0.399	0.407	0.382	0.397	**0.376**	**0.392**
	720	0.467	0.452	0.440	0.429	**0.431**	**0.421**
	avg	0.389	0.401	0.365	0.388	**0.364**	**0.385**
ETTm2	96	0.177	0.257	0.167	0.256	**0.165**	**0.254**
	192	0.240	0.298	**0.221**	0.293	**0.221**	**0.292**
	336	0.301	0.340	0.279	0.330	**0.276**	**0.328**
	720	0.401	0.398	0.374	0.387	**0.366**	**0.385**
	avg	0.280	0.323	0.260	0.317	**0.257**	**0.315**
Traffic	96	0.490	0.311	**0.419**	**0.303**	**0.419**	0.305
	192	0.486	0.307	0.427	**0.305**	**0.425**	0.308
	336	0.502	0.318	0.438	**0.307**	**0.434**	0.313
	720	0.537	0.335	0.472	0.329	**0.469**	**0.327**
	avg	0.504	0.318	0.439	**0.311**	**0.437**	0.313
Weather	96	0.162	0.209	0.151	**0.203**	**0.150**	0.208
	192	0.210	0.253	0.195	**0.246**	**0.194**	0.248
	336	0.265	0.293	0.249	0.288	**0.244**	**0.287**
	720	0.343	0.344	0.329	0.340	**0.315**	**0.337**
	avg	0.245	0.275	0.231	**0.269**	**0.226**	0.270
Electricity	96	0.157	0.251	0.137	**0.234**	**0.136**	0.235
	192	0.169	0.262	**0.156**	**0.250**	0.158	0.255
	336	0.188	0.280	0.173	0.269	**0.171**	**0.268**
	720	0.229	0.317	**0.208**	**0.298**	0.222	0.316
	avg	0.186	0.277	**0.169**	**0.263**	0.172	0.268

The best results are in bold.

**Table 6:** The MSE of the prediction results of FCP-Former and PatchTST under different patch lengths in the ETTh1 dataset.

Method	FCP-Former					PatchTST				
Patch Length	16	24		32		16	24		32	
Metric	MSE	MSE	|ΔMSE%|	MSE	|ΔMSE%|	MSE	MSE	|ΔMSE%|	MSE	|ΔMSE%|
96	0.378	0.379	0.26%	0.381	0.79%	0.378	0.389	2.91%	0.392	3.70%
192	0.426	0.426	0%	0.432	1.41%	0.443	0.451	1.81%	0.452	2.03%
336	0.472	0.482	2.12%	0.479	1.48%	0.493	0.508	3.04%	0.507	2.84%
720	0.471	0.471	0%	0.476	1.06%	0.527	0.542	2.85%	0.585	11.01%
avg	0.437	0.439	0.46%	0.442	1.14%	0.46	0.473	2.83%	0.484	5.22%

ΔMSE% represents the percentage change in the MSE value compared to the case with a patch length of 16.

**Table 7:** The MAE of the prediction results of FCP-Former and PatchTST under different patch lengths in the ETTh1 dataset.

Method	FCP-Former					PatchTST				
Patch Length	16	24		32		16	24		32	
Metric	MAE	MAE	|ΔMAE%|	MAE	|ΔMAE%|	MAE	MAE	|ΔMAE%|	MAE	|ΔMAE%|
96	0.395	0.396	0.25%	0.399	1.01%	0.395	0.402	1.77%	0.405	2.53%
192	0.421	0.423	0.47%	0.426	1.19%	0.435	0.442	1.61%	0.445	2.30%
336	0.445	0.448	0.67%	0.449	0.90%	0.461	0.468	1.52%	0.469	1.74%
720	0.460	0.462	0.43%	0.468	1.74%	0.499	0.501	0.40%	0.525	5.21%
avg	0.430	0.432	0.46%	0.435	1.16%	0.447	0.453	1.34%	0.460	2.91%

ΔMAE% represents the percentage change in the MAE value compared to the case with a patch length of 16.

**Table 8:** Results of robustness experiment in ETTh1 dataset.

Forecast Length	96	192	336	720
Metric	MSE	MSE	MSE	MSE
1	0.379	0.435	0.480	0.473
2	0.380	0.426	0.472	0.471
3	0.380	0.434	0.472	0.477
4	0.376	0.424	0.474	0.484
5	0.377	0.429	0.476	0.485
6	0.380	0.426	0.480	0.473
7	0.379	0.426	0.478	0.484
8	0.379	0.427	0.472	0.469
9	0.384	0.427	0.460	0.460
10	0.378	0.429	0.482	0.473
90% confidence bands	[0.377, 0.380]	[0.426, 0.429]	[0.471, 0.479]	[0.471, 0.478]
robustness	√	√	√	√

“√” indicates that the forecast results shown in Table 3 fall within the confidence bands.

**Table 9:** Results of the training cost experiment.

Methods	ETTh1						
	Iter	MSE	MAE	GPU	Epochs	TSPE	TRT
FCP-Former	22.7	**0.371**	**0.391**	1702	10	1.5	15
PatchTST	19.1	0.378	0.395	1696	**6**	1.26	7.56
iTransformer	**8.5**	0.385	0.404	**770**	7	**0.57**	**3.99**
TimeXer	18.4	0.386	0.399	1352	14	1.23	17.22
FEDformer	146.6	0.388	0.425	4798	12	9.82	117.84
Crossformer	57	0.384	0.408	3936	**6**	3.82	22.92
Autoformer	68.2	0.447	0.451	5298	**6**	4.57	27.42

The best results are in bold. Iter: Time spent per iteration (ms/iteration). MSE: Evaluation results. MAE: Evaluation result. GPU: GPU usage (MiB). Epochs: Number of training epochs. TSPE: Time spent per epoch (s/epoch). TRT: Total running time (s).

## Data Availability

Data available in a publicly accessible repository. ETT datasets: https://github.com/zhouhaoyi/etdataset (accessed on 10 January 2025), Traffic datasets: https://dot.ca.gov (accessed on 10 January 2025), Weather datasets: https://www.bgc-jena.mpg.de/wetter (accessed on 10 January 2025), Electricity datasets: https://archive.ics.uci.edu/ml/datasets/ElectricityLoadDiagrams20112014 (accessed on 10 January 2025), ILI datasets: https://gis.cdc.gov/grasp/fluview/fluportaldashboard.html (accessed on 10 January 2025). Further inquiries can be directed to the corresponding author.

## Abbreviations

The following abbreviations are used in this manuscript:

FCP-Former	Frequency Compensation Patch-wise TransFormer
RNN	Recurrent Neural Network
LSTM	Long Short-Term Memory
DFT	Discrete Fourier Transform
FFT	Fast Fourier Transform
TSPE	Time Spent Per Epoch
TRT	Total Running Time
